# The joys and frustrations of life as an embedded researcher: Findings from a diary study of researchers embedded within local authority public health teams in England

**DOI:** 10.1371/journal.pone.0328996

**Published:** 2025-07-28

**Authors:** Claire Stansfield, Dylan Kneale, Rachael C. Edwards

**Affiliations:** Evidence for Policy and Practice Information and Coordinating (EPPI) Centre, UCL Social Research Institute, University College London, London, United Kingdom; Simon Fraser University, CANADA

## Abstract

Embedded researcher interventions endeavour to facilitate culture changes in the production and use of research, so that research is contextually relevant for informing local policies and practices. However, the daily life of embedded researchers is poorly understood, as is their perceptions of working in a role intersecting research with local government settings. An embedded researcher intervention across English local authority public health teams provided an opportunity to understand what happens within these roles and which activities are undertaken. We describe the findings from a daily diary survey completed by embedded researchers for one week over two waves, comprising open-ended and closed questions (Nine participants in wave 1, of which five completed wave 2). Free-form entries were thematised using template analysis and quantitative data analysed through descriptive statistics. The diaries provided a valuable snapshot into a varied working life. Activities included: attending meetings; organising or requesting information; presenting evidence and data-preparation; collecting or analysing data; and providing information or research expertise. Embedded researchers require strong relational skills to collaborate with stakeholders in academia, community, local government and research funders on a range of topics. Participants reported both feelings of satisfaction and disappointment, and some described isolation. There was positive expectation that their activities gradually enhance useful public health research, though require time and system changes. Embedded researchers require support from stakeholders at all levels to maximise the potential benefits of their roles.

## Introduction

### The context of embedded researcher interventions to support local policies and practices

Embedded researchers are thought to be a promising approach for enhancing the production and use of research within policy and practice settings across a range of public and commercial sectors. [1–4]. They are theorised to bridge organisational siloes and to address gaps between research production and research engagement and use, with the intention of making research more beneficial and accessible to inform decision-making [1,2,5,6]. This is facilitated by embedded researchers being co-located across organisations. They may work ‘inside host organisations as members of staff, while also maintaining an affiliation with an academic institution’ [7,8]. However, variations exist around how co-location and co-affiliation are operationalised in practice, particularly given the growth in remote working arrangements since the COVID-19 pandemic [3].

There is growing interest in utilising embedded researcher roles within local public health contexts to support leaders in developing evidence informed local policies and practices for improving population health [3,6,9,10]. Within England, local authorities are responsible for planning and commissioning most local public health services. These services and measures are intended to improve the health and wellbeing of local populations, reduce health inequalities, ensure health protection functions are in place, and provide healthcare advice to the population. Services include those that are mandated (e.g., provision of sexual health testing services), and others that are discretionary depending on local public health needs (e.g., smoking cessation services). Decisions are made by elected officials, who are supported by practitioners and influenced by politics and public perceptions [11]. Research evidence that influences their decisions needs to be useful, accessible and overcome the barriers of dissemination, interpretation and implementation between academic research production and research use [12]. There is a need for public health evidence to have relevance to local populations, which may differ from national-level evidence and policies [13]. Co-produced research with stakeholders is encouraged so that evidence is more aligned with practice [14]. Embedded researchers can be viewed as embodying a form of co-production, where research is conducted *in-situ* with potential users [2]. However, other modes of the role exist and they might also influence and facilitate research conducted by others, or enhance the use of research in decision-making [3]. For example, embedded researchers may be the designated adviser on undertaking and using research [5,9], or undertake activities to build an organisation’s capacity to conduct, influence or use research [8,15].

We previously identified 35 evaluations of embedded researcher interventions in public health settings published between 2000 and 2021 [3]. These included evaluations of researchers embedded into practice (or policy) settings and, less commonly, evaluations of practitioners or policy-makers embedded within research settings. For example, health policy-makers in a regional government being seconded into a university [16]. The UK context was the most represented country setting (with 13 evaluations), followed by Australia, Canada and the USA (5–6 each). While the intervention is difficult to define [3], has wide variation, and there is limited understanding of how it is implemented in practice [17], there are nevertheless some common activities undertaken and mechanisms enacted that support the aim of bridging the gap between research and policy across diverse decision-making contexts. While the focus of the research presented in this study is in England, the processes described below are likely to have broader cross-country salience.

### Conceptual framework to understand embedded researcher interventions

Our earlier work [1,5] developed a framework to describe common processes and broad outcomes of embedded researcher interventions through six elements, drawn from mixed-method primary and secondary research. This is summarised as follows:

1) The intervention begins before a researcher is actually embedded, through the development of relationships between the organisations affiliated with the intervention (e.g., a policy or practice setting and research organisation) in order to co-create the role between stakeholders. Co-creation is not necessarily with the embedded researchers themselves.
2) An embedded researcher’s initial duties involve undertaking activities to maximise their visibility and help them become an insider within the host organisation.
3) Embedded researcher activities may involve a balance of conducting, brokering, and facilitating research. ‘Conducting research’ is understood as activities that directly involve the production of research; ‘brokering research’ is understood as activities involving the gathering, using and interpretation of research to inform decision-making; and ‘facilitating research’ is understood as activities that support others to lead or and conduct research.
4) Early and incremental successful changes include the development of networks and infrastructure, such as improved collaborative networks and recognition of the embedded researcher as a local advocate of research. These represent important precursors to cultural changes of organisations around conducting, brokering and facilitating research.
5) Longer-term impacts and signs of cultural change include instances where research is conducted (or commissioned) in a way that is more applicable to the stakeholder context and is used to inform local decision-making.
6) Although generally not observed in the literature, longer term systemic changes are theorised to follow, leading to strengthened cultures of evidence informed decision-making and, ultimately, improvements in population health.

The above trajectory is moderated by a range of contextual factors as well as the individuals within the embedded researcher roles. The potential for one person to achieve such culture change is limited within the large and complex remit of local population health within the context of societal challenges (e.g., the COVID-19 pandemic, austerity to public services). In a newly-created embedder researcher role, both role holders and the public health teams they work with must navigate ambiguity of tasks and performance expectations, and together understand the issues that research might be able to address before appropriate actions are taken. Furthermore, while ambiguity can provide autonomy for tailoring embedded researcher roles to individual contexts, skills and interests [18], it may bring challenges to roleholders working in isolation [18] and navigating their professional identity [19–21]. Moreover, the challenge of working within a position that has ‘culture change’ as its long-term goal is likely to create substantial workplace and wellbeing challenges.

### Study aims

Previous research investigating *how* embedded researcher interventions are implemented in public health, describe some activities that are undertaken though do not probe into *what* activities they undertake on a daily basis [2,6,22]. Allowing embedded researchers the space to record and reflect on their experiences could provide valuable insight into their daily activities and experiences and expand our understanding of such interventions. Using evidence from a diary study we aimed to understand: 1) how embedded researchers intersecting academia and policy and practice spend their time; 2) how these activities and achievements map to our conceptual framework of how such roles enhance the production and use of research; and crucially 3) how the embedded researchers perceive the importance of the work that they are conducting and the milestones they achieve.

### Study context

In this paper, we explore the daily activities and perceived challenges and enablers of embedded researchers within public health local authority settings in England. This study was a component of a mixed-methods research project that examined the influence of embedded researchers within public health settings [1]. The embedded researchers involved in this study were supported by the National Institute for Health and Care Research (NIHR) Clinical Research Network (CRN) and were embedded within English local authority (LA) public health teams. All posts were expected to contribute to enhancing research production and use within public health teams, although the design of the role differed across settings depending on the needs of the LA and on the needs of the NIHR and other organisations involved (e.g., universities). We refer to these embedded researchers as Public Health Local Authority Research Practitioners (PHLARPs), and use the terms PHLARP and ‘embedded researcher’ interchangeably in the remainder of the paper. Two began their posts in March 2020, and the remaining cohort started during spring 2021. The posts were initially for one year, though typically were extended by up to three years. Their posts were developed locally and across English regions. Physical placement and line-management varied between local authorities, regional Clinical Research Networks (CRNs) and Universities [1]. Although the intervention was implemented heterogeneously, the use of a diary study will help us to understand heterogeneity in daily practice.

## Methods

### Ethics approval and consent to participate

The study was reviewed and approved by the UCL IOE Research Ethics Committee (REC 1540) and all participants gave written informed consent.

### Sample

All PHLARPs were invited to take part in the study including through an online PHLARPs network event, and email invitations. A £50 incentive was offered on completion of the diary study for a week (5 daily entries; although these were occasionally spread across weeks depending on the working pattern of the embedded researcher). Nine participated in wave 1 (including one pilot participant), and they all were invited by email to wave 2, with non-response followed up at least once. Five participated in wave 2. Two participants had left the role before the follow-up study. Most participants reported being predominantly based in a local authority public health team, although two reported being affiliated to local authority teams while being predominantly based in a university (these could be considered to be embedded remotely, see [3]); all were affiliated with a regional CRN (seven CRNs were represented across the 9 participants in wave 1, and four in wave 2).

### Data collection

Diary studies are gaining prominence for collecting data on behaviours, experiences and perceptions near the time they took place and allowing sufficient detail of recall [23–25]. They are particularly useful for data that is difficult to observe and enables comparison at differing timepoints [25]. However, diaries rely on commitment and input of the participants [25]. Diaries have flexibility to capture different types of data, for example, qualitative data on work activities [25] and quantitative data on time use [26]. Challenges to qualitative data capture include the varying detail of responses, potential for under-reporting by participants, and labour-intensive data analysis [27]. In quantitative data collection there are challenges of interpretation of pre-specified categories by participants and having a lack of depth of data [26].

In this study, a one-week online diary survey (via Qualtrics) was used for mixed-method data collection and aimed to balance sufficient data capture without becoming too onerous on participant’s time. It was undertaken in two waves during 2022 (May to August) and 2023 (April to May) following piloting on one participant (the first wave had a longer timeframe to boost participation). Participants were provided with an information sheet describing the purpose of the study, the expectations on the time they should allocate to completing the study, and brief instructions on the content expected within their response next to the diary questions (see S1 Appendix). Participants were encouraged to fill in an online survey towards the end of their working day for 10–15 minutes for five consecutive working days, with an additional 10 minutes for the last day. They were informed that their responses would be confidential and anonymous and that their words would only occasionally be quoted to support explanations of our findings. The study information sheet included a weblink to resources on managing work stress in case participation raised anxieties about work.

The diary comprised closed and open-ended questions about the work activities undertaken during the day, feelings about the day and any informal connections they had with local authority colleagues. Additional questions at the end of the week prompted reflections on time spent on different activities, planned work that had not been achieved, interactions with local authority colleagues and how participants viewed their job role. The formatting of the second survey was slightly adjusted to prompt provision of more information around working activities, and on the enablers and challenges of the job role. Closed questions were asked as a way of prompting participants to recall tasks and feelings about their day, prior to open-ended questions requiring examples and descriptions of activities, informal connections and feelings. Each day participants were asked to indicate which activities they had been doing in their job role, and there was a choice of nine responses, including ‘other’ (the categories were developed by the research team). At the end of the week, they were asked to estimate the portion time spent on each activity, with totals summing to 100 and using the same nine responses. Each day participants were asked how much they agreed with statements about their day (1–5 scale): familiarity with the activities they encountered; high interest in work; high time pressure; encountering conflict with others. The daily open-ended questions asked participants to describe in greater detail (i) up to two activities, by providing a description of the activity, purpose and context; (ii) informal or social connections with colleagues and their perceptions of these; and (iii) how they feel about their day. At the end of the week, they were asked to reflect on their activities, interactions, and perceptions of their role.

### Data analysis

Analysis was undertaken in Excel and NVivo 14 by one researcher (CS), with checking by a second researcher (DK). Quantitative data was analysed through means, frequencies and comparisons. Qualitative data were thematised using template analysis (King 2012), which involved iteratively developing a coding template based on an initial set of 4 diary entries from wave 1 and applying this to subsequent diary entries in wave 1 and reviewing the codes again (supported by a descriptive codebook). One researcher (CS) developed the initial template and applied the codes. Two researchers (DK, RE) each tested the template on two different diary entries, and provided input to the final template. The template was also informed by collective discussions of findings across the wider research project. Wave 2 entries were coded to the template from wave 1. Brief sketches were developed of each participant to aid analysis of themes, and support comparisons of responses at the two time-points. Template analysis is a technique to provide a structured approach to data handling (King 2012) and is based on developing a set of codes from analysing a subset of data, which are used as a template for further coding and analysis. The codes are modified as analysis progresses further (Howard et al. 2008).

## Results

The results are presented under six areas: i) an overview of a working week (study aim 1); ii) activities of significance to embedded researchers (study aims 1 and 3); iii) activities that illustrate the processes of becoming embedded and remaining embedded (study aim 2); iv) the complex balance of activities related to conducting, facilitating, brokering and using research (study aim 2); v) early gains in shifting organisation culture towards research (study aim 2); and, vi) the joys and frustrations of being an embedded researcher (study aims 1 and 3).

### A week in the life of an embedded researcher

Embedded researchers (PHLARPs) were provided with a framework for recording distinct types of activities, and the results underscore that each day is different, and most undertook a variety of different activities throughout the week. Table 1 shows that attending meetings was an activity undertaken by all embedded researchers on most days, and on average accounted for over a quarter of their time (26%; ranging from 10–60% of time spent in a week). Other common activities that embedded researchers spent an average of 10–15% of their time on included presenting evidence and related preparation activities, providing information or research expertise, requesting information, organising information and collecting or analysing data (range 2–40%). Self-learning, training others and other activities were less common and comprised 1–6% of the time (range 0–13%). Table 1 also shows that the average time spent on various activities were broadly similar for both waves.

**Table 1:** Activities undertaken over the week.

	Daily reporting of activities undertaken				Time spent on different activities over the week (%) (end of week reporting)			
**Activity**	**Wave 1 (N = 9)**		Wave 2 (N = 5)**		**Wave 1 (N = 9)**		**Wave 2 (N = 5)**	
	**No. of participants undertaking this during the week**	**Range: Days per participant over 5 days**	**No. of participants undertaking this during the week**	**Range: Days per participant over 5 days**	**Mean**	**Range**	**Mean**	**Range**
Attending meetings	9	3-5	5	4-5	26	10-42	33	22-60
Presenting evidence (verbal or written reports), including preparation activities	8	0-5	5	1-3	10	2-19	10	8-12
Providing information or research expertise	8	0-5	5	2-3	13	2-20	14	4-32
Requesting information	7	0-5	3	0-2	11	0-31	10	1-15
Organising information (filing, sorting or checking)	7	0-5	3	0-2	15	6-39	4	0-10
Collecting or analysing data	6	0-4	3	0-5	13	2-35	16	5-40
Self-learning activities (formal or informal)	6	0-2	4	0-2	5	0-11	6	0-13
Other^*^	5	0-3	1	0-1	1	0-10	3	0-10
Training others	4	0-2	2	0-1	5	0-13	4	0-11

* Wave 1: Other: travelling and attended a conference; email and diary management; follow-up work from the meetings; general discussion with colleagues on work; planning events and workstreams; providing support to research staff in other local authorities; setting up meetings; not specified.

Wave 2: Other: attending a conference.

** 1 day of missing data for one participant.

### Activities of significance for embedded researchers

The following eight themes of activities were developed from the participants’ responses when prompted to describe up to two activities: building linkages; applying for funding; planning research; public engagement; undertaking research or supporting research use; supporting skill development; self-development; and general administration (including time to catch up and general team meetings). These themes are described with examples in Table 2. Unlike the data presented in Table 1, which were collected through a pre-determined framework, these data allow us to examine activities of significance for embedded researchers. However, it was not always possible to determine the activity undertaken by the PHLARPS from their descriptions; activities that were planned and not undertaken that week also contributed to the themes. Where meetings were described by participants, we tried to code these by the type or purpose of the activity. For example, a meeting discussing early ideas for a funding bid, was coded as “applying for funding”

**Table 2:** Key activities described by PHLARPs and example statements.

Theme	Respondents [n = 9]	Examples
Building linkages	9	“Speaking to local universities about potentially teaching/speaking to students on research in local authorities and NHS”
Applying for funding	7	“Writing a first draft proposal for a NIHR funding call that will speak to the research interests of council colleagues and […] academics who have expressed an interest in being involved”
Planning research	7	“Preliminary scoping exercise to determine what data we hold on [….] and how we can link this to […} and other datasets”
Public engagement	6	“preparing some community insight work…. included listing locations that would be beneficial…..along with tips for speaking ….[to] the general public”
Undertaking research/supporting research use:		
i) Data collection, collation and analysis	6	“Continued data analysis on a set of interview transcripts from[…..] feasibility study”
ii) Data governance	3	“regular meetings with data analysts, experts and governance specialists”
iii) Promoting research use by enabling or encouraging others	4	“Liaising with local universities, academics and researchers on public health research and potential uses of existing research and prepared datasets for [..]”.
iv) Other activities to support data or research use	4	“Strategic meeting to ….”plan for future council-based opportunities to use [] research findings to inform policy making”
v) Other research facilitation activities	6	Diarist met with a LA director to secure a funding commitment for an academic to pilot an intervention. “I attended a ‘research priority setting’ meeting”, “meeting with two collaborators from [named University] to discuss the target for a study”
vi) Other research production activities	7	“[Meeting] to present and disseminate the findings of a recent NIHR funded project…. followed by questions and discussion regarding our data and what the next research step will be”
Supporting skill development	8	“Meeting with fellow data analysts to help them develop data linkage projects” “identifying existing suitable training for LA staff”
Self-development	4	“Attending the [] Conference - learning, networking, spreading the word of my role”
General administration (including time to catch up and general team meetings)	9	“30 mins opportunity to catch up with what colleagues across [public health] have been doing and allow for cross-pollination”

In terms of the most common activities, building linkages is a key activity taking a number of forms (described later in the text). Most of the nine participants provided details about attending a team meeting during the week (n = 8) and provided training or support to others (n = 8), of which half also enabled others to use research (n = 4). (Data corresponds to a participant mentioning this one time or across either wave). A majority described undertaking an activity related to research funding (n = 7) and planning research (n = 7), and other activities related to research facilitation and research production (n = 7). Six reported involvement in public engagement activities. Other activities described by fewer than half include activities to support data or research use, data governance, data preparation, and self-development activities.

Our analysis of these activities (Table 2) compared with the stages within our conceptual framework shows the that embedded researchers were (i) undertaking activities to maximise their visibility and become embedded (stage 2); and (ii) undertaking a complex balance of activities around the generation, brokering and facilitation of research (stage 3); but also (iii) were operating in a highly relational role where early gains were being observed in the steps towards involvement in research production and use (stage 4). We describe these in further detail below.

The roles were also observed to be working across a wide variety of public health initiatives from those related to the social determinants of health (e.g., early years development and learning, housing, and food banks) to those on particular health conditions (e.g., social isolation, dementia and obesity)

### Becoming embedded and addressing needs

The choice of activities described in Table 2 underlines the importance not only of activities that could be considered core to the role (conducting and facilitating research and research use), but illustrate the diffuse process of becoming embedded. Despite having been in post for at least a year, several activities appear to involve a PHLARP’s engagement with colleagues and networks to establish the nature of their embedded researcher role (see later section) and to understand the culture of the local authority. For example, while we might expect a needs assessment/situational analysis to be conducted in the early stages of ‘becoming embedded’, analysis of diary entries revealed that assessing needs was a more continuous process than a discrete starting point. The PHLARPs described supporting skill development through identifying training needs of LAs, teams or individuals. This could be from established meetings, for example a quarterly meeting to discuss training and support, through to more discrete activities. However, it was also clear that the PHLARPs progressed beyond *assessing* needs to *addressing* needs through, for example, identifying or organising training activities such as webinars, creating guides for data analysts and researchers, and updating online resources to raise awareness of training. Capacity building took the form of sharing knowledge among peers and colleagues, providing support for those new to research. This could be informal or developed strategically: *“The meeting helped identify a mutual objective in identifying what training/learning opportunities already exist and what gaps are needing to be bridged so research delivery can be further enhanced.”*

### Undertaking a complex balance of activities around conducting, facilitating, brokering and using research

#### Conducting research.

The PHLARPs reported directly undertaking data collection and analysis activities commensurate with conducting research, although these were described with the purpose of feeding into strategies or supporting further research. Example activities included preparing and linking datasets for internal and external use, gathering and reviewing prevalence data of disease and public health indicators, scoping the data that existed in an area and linking to other datasets, or analysing interview transcripts: “*Continued data analysis on a set of interview transcripts from[…..] feasibility study.*” Conducting research was not frequently described in detail by the PHLARPs, perhaps reflecting that this was not an activity that most PHLARPs were either heavily engaged in, or because they were closely facilitating others leading the research.

#### Research facilitation.

Much of the PHLARP role involved facilitating research rather than directly carrying out research, although conducting research and research facilitation could not always be considered discrete activities in practice. For example, activities within the theme of ‘planning research’ included developing study protocols and assisting with participant recruitment, and involved a blend of research facilitation and conducting research. Other examples included meetings to discuss project plans, designing a questionnaire for an evaluation, initiating an advisory group, discussing the focus of a study and clarifying project delivery goals. Planning research also involved building linkages and working with local authority staff, elected council members, academics and community organisations in some cases: *“…the opportunity to finally meet up face to face with a newly elected cabinet member-to discuss a pilot […..] research protocol that I’m developing and piloting in [….].*”

Several PHLARPs appeared to be engaged in activities to support data linkage and data governance (i.e., research facilitation) which perhaps speaks to a broader concern across local authorities to maximise opportunities for the use of data to inform decision-making. Data governance was mentioned in several contexts, including meetings attended by governance specialists for particular projects, obtaining copies of data protection documents, completing a data protection impact assessment, and participating in a team information session on new data collection and storage processes within an LA: “*Liaising between legal departments [] and external research partner [] to move forward the legal agreements underpinning a specific research project*”.

#### Brokering and using research.

There were examples where existing research was identified and analysed (e.g., brokered) and used to inform decision-making. However, as exemplified by one PHLARP, this often appeared to involve drawing on LA data and government and other reports rather than academic research presented in journal articles: “*Writing up an action plan for the borough for suicide prevention by looking at data and reading other reports to make our own borough data*”. Other linked activities included supporting peers through providing guidance on interpreting a particular research study, as well as broader communication activities. Other activities promoting research use included creating a webpage for the region to support public health research in local authorities, and identifying ways to communicate research findings within a local authority, as one PHLARP discussed in their (thwarted) plans: *“I had hoped to explore ways of more effectively presenting research finding briefing papers to council colleagues, but didn’t have time”.*

### Early gains in shifting organisational culture towards research

Three themes of activities are described here that demonstrate early and incremental gains in shifting organisational cultures towards research: Championing research across multiple stakeholders; building links and maximising visibility as core activities and processes; and developing infrastructure to support new research activities.

#### Championing research across multiple stakeholders.

Having local advocates of research is an important precursor for activities to promote culture change in conducting, facilitating and using research. The diary study shows that PHLARPs had roles in championing research both within the local authority and with the wider public. The PHLARPs described activities that aligned with becoming recognised research experts within LA teams, and informing decisions about which forms of research the LA should engage in, for example: “*Reading a protocol and getting new information about a new study to see if it suits the local authority*”. Public engagement activities ranged from project planning for engagement with the public; promoting research at an event attended by residents and patients, community and voluntary sector providers; running a public engagement workshop; and applying for funding to develop public engagement events with underserved communities. These activities also included setting up the conditions to maximise public involvement through, for example, developing a rewards and recognition payment policy for public contributors, as well as “*identifying the appropriate language for researchers and local authority members to use with members of the public when engaging them in research*”.

#### Building links and maximising visibility as core activities and processes.

The diary responses clearly show building linkages with others is a key part of the PHLARP role, with PHLARPs reporting efforts to develop links with local authority and Clinical Research Network staff, local councillors (local elected representatives), researchers in academia, community stakeholders, practitioners, the public, as well as peers doing similar roles. Activities included participating or developing in networks, meeting individuals and teams, and public event attendance. The PHLARPs linked people together to inform, develop, undertake or use research: *“Being embedded within the public health division provides me with a cross-organisation (and wider partnership) perspective that enables me to connect and engage with relevant partners within the council and across the wider partnership. This week this has enabled me to connect local subject matter experts (from the council and from external health partners) with external academics in order to bring their expertise into new research, and has enabled me to bring the right ‘operational’ colleagues together with researchers to ensure that the delivery of an established research project happens effectively.”*

Building linkages took a range of forms and was conducted for a number of different purposes, including:

- **Individual connections:** Connecting with individuals to meet, foster and understand mutual areas of interest for current work and future opportunities. These connections may be both formal and informal developed from planned meetings, following up email enquiries, conference attendance, event attendance (e.g., project launches).
- **Team building and sustaining embeddedness**: Linking within team or immediate working environment. This could be from participation in staff meetings and team events, or through individual catchups and advice, working in the same office environment. For example, team meetings may help “*better cross-pollination of ideas*”, improves their understanding of needs to link relevant people together or find out what is happening within LA, or raising awareness within these teams to services and opportunities.
- **Network development:** Participating and developing in a variety of networks, from community forums, topic-focused forums, and multi-agency meetings related to public health research activities, peer networks, and through project-related activities.
- **Mobilising external expertise**: Targeted linking of people, projects or services, such as identifying academics who can support a particular project to advise council, liaising with academics, encouraging research contacts to feed into a review, or “*speaking to local universities about potentially teaching/speaking to students on research in local authorities and NHS*”
- **Maximising visibility**: Maximising the visibility of LA engagement with research activity through the above forms of linkages and written communications, e.g., newsletter piece promoting a research initiative, and a contributing to a regional website raising awareness of public health research events, opportunities and training.

Networking activities also provided the PHLARPs opportunities to realise differences that they were beginning to make in their role: “*very positive feedback from discussions/meetings within academia about how my role has actually facilitated a lot of opportunity that hasn’t happened elsewhere*”.

Developing infrastructure to support new research activities: Several activities involved securing resources to undertake further research. Funding opportunities sought by the PHLARPs included those from the UK National Institute for Health and Care Research (NIHR) funding calls, a university scheme and local authority funds to support a pilot study. Activities related to applying for funding included meetings to develop bid ideas, engaging with potential or existing collaborators (within a local authority, academics, community organisations), writing a bid, preparing costings, and collating and submitting feedback to funding reviewers. It also included writing briefs about funding calls for others. Submitting funding proposals appeared to be an activity that embedded researchers engaged within once they felt established in post: “*The more I engage in regular contact with local authority staff the more my confidence grows in engaging in the co-development of bids and research projects*.”

### Joys and frustrations of the embedded researcher

Challenges, enablers and feelings encountered by the PHLARPs are described here within the themes of: In-person and remote working, the ambition of the role and daily joys and frustrations.

#### Embeddedness and remote working as challenges and enablers to building linkages.

Developing contacts with those working in a local authority, academia or with other stakeholders was reported to be a challenge in some cases. While most PHLARPs were based within a local authority, a smaller number were based in a university and worked with LAs at a distance, and at least one worked across a range of LAs. Some PHLARPs described the challenge of influencing cultures while having low levels of embeddedness within a LA: “*One of the biggest challenges I’ve encountered is actually being embedded within a university. Although I have developed a great working relationships with Local Authorities, often I find that it is hard to influence research decisions within Local Authorities.*”

The PHLARPs had to navigate remote, online working whichever type of host organisation they were based within (e.g., working at home themselves or have colleagues who worked at home, which was particularly common following Covid-19 lockdowns and their aftermath). Remote working was viewed by some as slowing the pace of building some relationships and gaining ‘soft’ knowledge. In the first wave of data collection (spring 2022), some participants found remote working to be a barrier to becoming embedded. They described having little contact with local authority staff which hindered maintaining connections, finding out what is going on, and the visibility of the role. A year on, in wave 2 this shifted to some extent (and likely reflected changes in organisational working practices that encouraged in-person office days). Having in-person days to connect with colleagues, and in-person events were appreciated by some. However, sometimes in-person working was restricted to certain teams (owing to limited office space), and there were less frequent connections where teams did not overlap. One participant instigated a regular online ‘meet-me’ for colleagues to improve visibility and connection. Moreover, it was observed that working online can facilitate and widen attendance of online meetings: “*Challenges encountered are probably due to remote working and not being able to build relationships as quickly. However, online meetings do tend to provide good attendance and a chance to speak to people who wouldn’t normally be able to be available due to time/travel commitments.*”

#### Matching the ambition of changing a research culture through the actions of an individual.

Some PHLARPs reported feeling a lack of influence on decision-making or challenges in engaging colleagues with research and maintaining their commitment, partly owing to the junior (early career) context of the role and also their capacity to address the large scale goal to embed research. They described issues with achieving the support necessary to orientate public health teams towards engaging with research as an individual, occupying an intersectional position, and often in an early stage of their career: “*It is challenging to initiate and sustain momentum around complex change, beyond the capacity of a single role, and it requires strategic and influential champions to drive forward.*” Conversely, the PHLARPs who reported ‘buy-in’ from senior colleagues perceived greater levels of influence, for example: “*The Director of Public Health is very engaged in this post - and this means […] I am able to access and create opportunities to promote work and engage with wider council/non-council structures*”

Some PHLARPs were experienced academic researchers, and while attempting to change cultures, diary entries also showed that they were also still grappling with cultural differences in ways of working and expectations between research and local authority needs: “*the differences in time frames, lack of research skills and knowledge in local government are real and remain. It certainly feels as though that has shifted in this local authority over the last two years however*.”

Despite being in the role for over a year at the first point of data collection, some embedded researchers felt that the aims of their role remained vague and that the role of the stakeholders involved was still being configured (most commonly the relationship between the funder and overseer of the embedded researcher intervention (NIHR-CRN) and the LA), which in some cases led to feelings of being underutilised or misunderstood: “*it is hard to know if we are achieving our targets when our roles are so vague*”.

By wave 2, two diarists who participated in wave 1 had already left their posts. It is unclear whether these moves were driven by uncertainty about future funding of the role, dissatisfaction, or new opportunities. Nevertheless, the diary entries appear to suggest that the role requires a long-term commitment from funders and stakeholders to enable the embedded researcher to build up a profile and maximise the visibility of the role: *“Over 2 years I have been able to build the profile and reach of this role….The role is critical towards fostering and developing a culture of research in local authorities-which is still in its infancy.”*

#### Daily joys and frustrations of the role.

The PHLARPS were asked to rate their daily feelings across a number of items (Table 3), and the daily feelings of PHLARPs were largely high familiarity and high interest with work, without being over-pressured and low conflict, though time pressure and conflict had greater fluctuations (as shown in the standard deviations). These observations were broadly consistent across both waves.

**Table 3:** Ranking of daily average feelings across five days (High = 5; Low = 1).

	Mean (Range, Standard Deviation)	
Wave	1 (n = 9)	2 (n = 5)
Familiarity with activities	4.1 (2-5, 0.79)	4.1 (2-5, 0.76)
Interest in work	4.4 (3-5, 0.72)	4.1 (2-5, 0.78)
Time pressure	2.4 (1-4, 0.91)	2 (1-4, 1.04)
Conflict with others	1.5 (1-4, 0.91)	1.4 (1-4, 0.76)

The diary narratives (open text entries) did not always correspond with reported agreements, particularly for time pressure and conflict. Within their diary narratives, all participants reported positive feelings of satisfaction or enjoyment, and most commented on feeling connected and supported (n = 8). However, the same number also reported some frustrations or disappointments and three described isolation or lack of connectivity in the role: “*Working within the LA is very frustrating. There is no connectivity and a lot of duplication going on….I feel there needs to be a culture shift*”. It was acknowledged within diary entries that creating change within public health teams would take time and needed wider support and system-level changes. Nevertheless, overall, there appeared to be a positive expectation that the activities of PHLARPs made an incremental contribution to enabling research production and use within LA public health teams: “*Really happy as people are genuinely enthusiastic about our work and the possibilities*”. Returning to the first sets of results, daily variation in the types of tasks undertaken appeared to be a core theme in the reflections of our embedded researchers, with descriptions of ‘busy’ days and comprised of ‘a range of activities’ frequently mentioned as components which helped to sustain interest in the role and ‘prevent burnout’, and as reflected by one embedded researcher: “*The variety involved is both hugely enjoyable but equally terrifying.*”

## Discussion

Diary data collected among embedded researchers show that their roles are characterised by substantial variation, and indeed ‘terrifying’ amounts of variation as described by one embedded researcher, on an ongoing and daily basis. The diary data nevertheless emphasises that this variation is an attractive and highly rewarding part of the role. Furthermore, diary entries underscore that a year into their roles, embedded researchers made important inroads in terms of creating a more research-active culture within public health teams. Several built strong networks and links to help facilitate and mobilise research, or became local internal- and external-facing advocates of research, or started to change the infrastructure of conducting research. This latter aspect speaks to the sustainability of the actions of the embedded researchers involved in this study. Embedded researchers linked their local authority public health teams into the broader research infrastructure as part of the process of facilitating and developing funding proposals for research and evaluation projects. Successful proposals attract resources for conducting research that involve, or are potentially led by, local authority public health teams.

However, the evidence here also shows that there is wide variety in the experiences recorded, beyond having ‘good’ or ‘bad’ days. Participants appear to have different expertise, experience and confidence, as well as differing levels of support. Related work analysing the job descriptions and recruitment packs for the roles included in the scheme shows the roles themselves as highly heterogeneous in set-up and in terms of the skill profile that was sought for the role [1]. However, the evidence in this diary study shows that in all cases, an embedded researcher intervention is a small, incremental, and complex intervention requiring time for developing and building linkages. Support and infrastructure are needed for the ambitious expectations of the role to be addressed.

Ward et al [17] grouped the activities undertaken by embedded researchers in UK health settings into four types: relational, knowledge creation, education, and project management. This categorisation also describes the PHLARPs activities; although in our study we have considered the function of embedded researchers (around research generation, brokering and facilitation) and described activities underpinning these. The findings here, and those in Ward et al [17], underscore that embedded researcher roles have a relational component at their core that is crucial for achieving culture change. Research based on embedded researcher roles in clinical health points to particular skillsets required for working across practice and policy environments [19–21,28], which in addition to substantive and methodological knowledge, in our study appear to include communication skills to working across cultures with people from a range of contexts; flexibility to undertake a variety of activities, and a willingness and ability to ‘manage upwards’ as an embedded researcher attempts to influence change within a relatively vague remit. These skills may change over time, and as the role and experience-level shifts [29], although in our study we observed comparatively little change over time. The focus of the diary was on which activities were undertaken and how the PHLARPs felt. These activities were undertaken against a background of potentially competing agendas of the local authority, the funders and overseers (NIHR and CRNs) and university employers, and of the personal agenda of the individual within the role. However, the breadth of public health topics and the range of stakeholders involved in shaping the role appeared to present few obstacles to embedded researchers. Instead, diary entries highlight that most embedded researchers could see the value of their role, although the data did indicate that the ambitions tied to the embedded researcher role could add to a sense of pressure and frustration at the pace of change at times.

### Strengths and limitations

Participants in the PHLARP scheme, and diary study, differed in the type of experience they brought to the role (such as research or practice or policy experience. A strength of this study is the insight gained into common activities and experiences that can exist among diverse embedded researcher roles. Further, linking broad types of activities and their purpose to a framework of how embedded researcher interventions are thought to work [1] also increases the applicability of the finding to informing similar roles within different contexts. Another strength of this study is the focus on the activities that were actually undertaken during two working weeks, at different timepoints and from the participants perspectives. However, the nature of a diary study means the data is self-reported, and participants differed in writing style, depth of description, and explanation for their responses. We interpreted the free-text responses and assigned to our coding template and others may have interpreted these differently (e.g., such as the type of research activity). At least one participant declined to describe any feelings when asked how they felt about their day, though at the end of the week shared challenges and benefits they identified around about the role. Some participants recorded brief entries that were uninformative for our analysis, for example, a two-word response of ‘*Hump day’* (a colloquial term for Wednesday). There were also gaps in the quantitative data on feelings. The quantitative data was collected mainly as a prompt to facilitate reflection by the participants prior to them providing their own descriptions. It also provided a way to efficiently capture data in a short time each day. However, occasionally there was little triangulation between the quantitative data and free text entries. For example, one respondent rated their interest that day as high but stated *“Felt okay today. Motivation to work was low”*, suggesting that the diary entries captured both professional and personal reflections. Some respondents could potentially feel cautious in how candid they could be in free text entries or be unclear on the level of detail to write. Furthermore, we didn’t provide any ‘training’ on completing the diary and relied on instructions presented in the diary itself. Participants could describe two activities undertaken each day, which could have been notable for a time, importance or another reason. Sample size was an issue with this study, and there was substantial attrition between both waves of the diary. Among those who completed both waves, there were few perceptible differences between the perceptions of the roles and activities undertaken; we cannot discount the possibility that this pattern would change had the full sample participated in both waves. We could not infer statistical significance from the quantitative data owing to the small sample size. We also asked participants to reflect on the process of completing a diary. Most comments on participation were favourable, and negative feedback was confined to IT issues. As one PHLARP reflected: *“It’s been a useful experience; it has always been the last thing that I’ve done of a day which I’m afraid may impact on the quality of response. But it’s a well-tailored way of getting insight. And it’s been rewarding for me also”.*

The researchers conducted this research as part of a work programme of researching embedded researchers generally, and specifically the PHLARP scheme [1] and interacted with an advisory group for the project, presenting interim findings at meetings]. We believe these experiences increase the credibility of our findings.

## Conclusions

This study provides valuable insights into the activities, benefits and challenges of implementing an embedded researcher model tailored to individual settings within English local authorities. However, we believe these insights are applicable to similar roles in other settings that aim to bridge and adapt research production and research use. The study also gives an indication into the profile of individuals who are engaged in embedded researcher posts, who appear to thrive on the variety and challenge of working in such a role. The diary entries emphasise that an embedded researcher role is a relational role where individuals draw on their interpersonal and influencing skills to advocate research, change the infrastructure for conducting research, and position local authorities within research-related networks. To better enable this, embedded researchers require support at all levels and from all stakeholders involved to maximise the potential benefits of their roles.

## Supporting information

S1 Appendix Diary instructions and questions. (DOCX)
