# Supplementary material for: The joys and frustrations of life as an embedded researcher: Findings from a diary study of researchers embedded within local authority public health teams in England
Source: PLoS One. 2025 Jul 28;20(7):e0328996. doi: 10.1371/journal.pone.0328996 (PMC12303328; doi:10.1371/journal.pone.0328996)
Supplement: S1 Appendix — (DOCX) [file pone.0328996.s001.docx]

# **Supporting information** Diary instructions and questions

The following pages show the instructions provided to participants for Day 5. On this day, the participants were provided with the same questions for days 1-4, plus an additional end of week survey.

1. Example introductory text;
2. Daily dairy survey;
3. End of Diary Survey;
4. Changes to Diary questions in wave 2.

**1. Example introductory text**

Thank-you for taking part day 5 of this diary study.  This is the last day of the study and has additional questions to the previous days. Please allow 10 minutes longer to complete this today. We would like you to answer all questions as fully as possible.   If you cannot answer, please skip the question or say 'not applicable' in the space provided. You can see a reminder of the information supporting this study here: [*Google* *form link*] and you can also take a look at the consent form you submitted to the researchers if you need to revisit this. If you experience any problems with this survey, please get in touch with [*contact details*]
Thanks again for taking part!

**2. Daily dairy survey**

Q1 Record the day for this diary entry

- Monday
- Tuesday
- Wednesday
- Thursday
- Friday
- Saturday
- Sunday

Q2 What activities have you been doing today in your job role?

- Attending meetings
- Collecting or analysing data
- Organising information (filing, sorting or checking)
- Presenting evidence (verbal or written reports), including preparation activities
- Providing information or research expertise
- Requesting information
- Self-learning activities (formal or informal)
- Training others
- Other ________________________________________________

Q3 Describe up to two of these activities.
Please include a description of the activity, the purpose and context.

For example:
Describe the activity: e.g. attending meetings/ analysing a report/ writing up a report.
Purpose of activity: e.g. what the meeting or report was for.
Context/how: e.g. who you were meeting (e.g. councillor)/ what is the report about.

There are separate boxes to record each activity. If you do not have an activity to describe, write 'not applicable'.

Activity 1
_______________________________________________________________

Activity 2

________________________________________________________________

Q4 Were you involved in any informal or social connections you have with colleagues today?   This might help us understand how your role is embedded into a team.

Describe what these activities were, and the roles of those you interacted with, or indicate 'none'.

For example, if it was an online or in-person coffee break, or informal conversations before a meeting.
The people might be a team member, colleague within the local authority, colleague from academia, external practitioner, mentor, peer from CRN-network or other.

________________________________________________________________

Q5 Describe anything that made you feel distant from your colleagues, or indicate 'none'.

________________________________________________________________

Q6 Move the circle to indicate how much you agree with the statements about your day

|  | Strongly disagree | Somewhat disagree | Neither agree nor disagree | Somewhat agree | Strongly agree | Not Applicable |
| --- | --- | --- | --- | --- | --- | --- |

|  | 1 | 2 | 3 | 4 | 5 |
| --- | --- | --- | --- | --- | --- |

| I was familiar with the activities I encountered in my day | 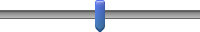 |
| --- | --- |
| I was highly interested in my work | 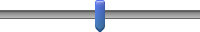 |
| I was under high time pressure | 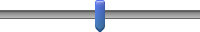 |
| I encountered conflict with others | 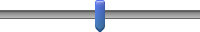 |

Q7 Describe how you feel about your day

________________________________________________________________

|  |
| --- |

**2. End of Diary Survey**

These questions prompt reflection of your last five working days.

Q8 Give an approximate indication of the portion of time you spent on each activity. 

Total values will need to sum to 100.

_______ Attending meetings

_______ Collecting or analysing data

_______ Organising information (filing, sorting or checking)

_______ Presenting evidence (verbal or written reports), including preparation activities

_______ Providing information or research expertise

_______ Requesting information

_______ Self-learning activities (formal or informal)

_______ Training others

_______ Other

Q9 Describe any activities you had planned to undertake but did not do so, and feel free to explain why.

________________________________________________________________

Q10 Describe your interactions with your local authority colleagues. 

What worked well and what challenges did you encounter? 

You can go into as much detail as you want and please remember that this diary will be kept confidential.

________________________________________________________________

Q11 Please use the space below to reflect on how you view your job role and anything else you think would be useful for us to know about your day-to-day work life.

________________________________________________________________

Q12 We plan to run a follow-up diary study at some point. You are welcome to comment on your experience of completing this diary and suggest improvements.

1. **Changes to Diary questions in wave 2**

The questions for Wave 2 were similar to Wave 1. It included an addititonal question "*What helps or hinders your job role?*" and did not include Q12. Prompts and spaces were added to help encourage fuller responses to the questions. For example, in Question 3, a prompt was added e.g. "*Activity 1*" was replaced with "*Activity 1 - Describe the activity, purpose and context*", and the original Question 10 had two separate spaces allowing separate responses for: "*What worked well?*" and "*What challenges did you encounter?*".
